# Supplementary material for: Cloud radiative effects significantly increase wintertime atmospheric blocking in the Euro-Atlantic sector
Source: Nat Commun. 2025 Nov 5;16:9763. doi: 10.1038/s41467-025-64672-9 (PMC12589630; doi:10.1038/s41467-025-64672-9)
Supplement: Supplementary file 1 — Supplementary Information [file 41467_2025_64672_MOESM1_ESM.pdf]

Cloud-Radiative Effects Significantly Increase Wintertime Atmospheric Blocking in the Euro-Atlantic Sector

Sandro W. Lubis[1,*], Bryce E. Harrop[1], Jian Lu[1,2], L. Ruby Leung[1], Ziming Chen[1], Clare S. Y. Huang[3], Nour-Eddine Omrani[4]
[1]Pacific Northwest National Laboratory, Richland, WA, USA
[2]College of Oceanic and Atmospheric Sciences and State Key Laboratory of Physical Oceanography, Ocean University of China, Qingdao, China
[3]Epsilon Data Management LLC, Irving, TX, USA
[4]Bjerknes Centre for Climate Research, University of Bergen, Bergen, Norway

Table S1 Summary statistics of wintertime (DJF) blocking frequency climatology from the CFMIP experiments in the Euro-Atlantic sector. The unit is the percentage of blocked days in the season (i.e., with 2% corresponding approximately to two blocked days per winter). An asterisk (*) denotes significant difference from CTL at 95% level.

Model	% (ctl)		% (lwoff)		Relative change (%)
	mean	stddev	mean	stddev	
CESM2	10.08	2.74	9.02	3.08	**-10.52***
HadGEM3-GC31-LL	10.64	2.63	8.23	2.54	**- 22.65***
IPSL-CM6A-LR	12.51	2.87	9.10	2.74	**-27.26***
MRI-ESM2-0	10.74	2.29	10.61	3.26	-1.21
MME Mean	10.99	2.63	9.24	2.91	**-15.41***

Table S2 Summary statistics of wintertime (DJF) blocking frequency climatology from the CFMIP experiments in the Western/Central Europe sector. The unit is the percentage of blocked days in the season (i.e., with 2% corresponding approximately to two blocked days per winter). An asterisk (*) denotes significant difference from CTL at 95% level.

Model	% (ctl)		%(lwoff)		Relative change (%)
	mean	stddev	mean	stddev	
CESM2	9.36	2.56	8.15	3.36	**-12.93***
HadGEM3-GC31-LL	8.98	2.87	6.60	2.80	**-26.50***
IPSL-CM6A-LR	8.81	2.14	6.18	2.22	**-29.85***
MRI-ESM2-0	9.53	2.22	9.10	3.15	-4.51
MME Mean	9.17	2.45	7.51	2.88	**-18.45***

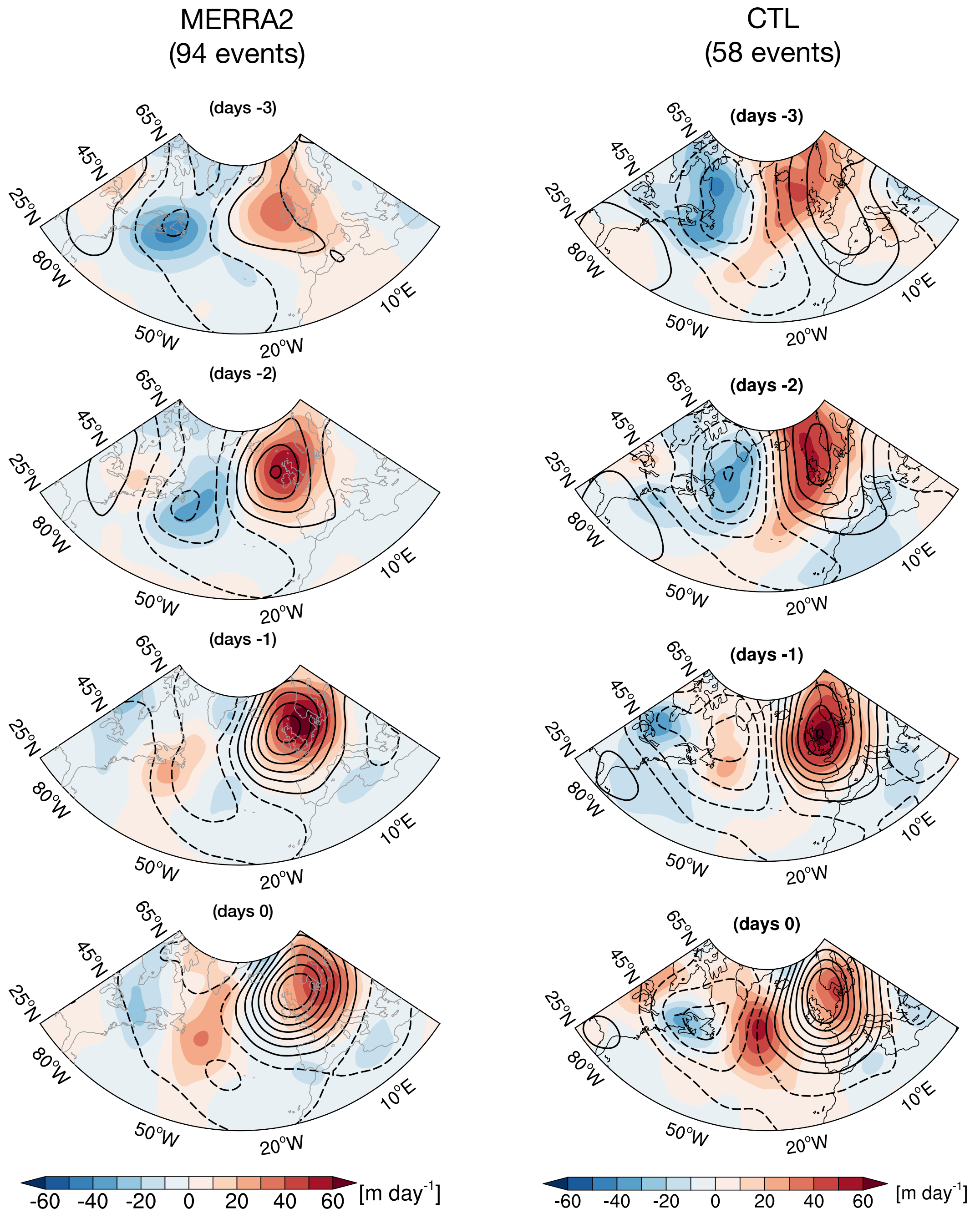

Fig S1 Ability of the E3SM atmospheric model to simulate the development of Euro-Atlantic blocking. Composite of the 500-hPa geopotential height anomaly (contours; interval: 50 m) and its tendency (shading; units: m day^{-1}) from days –3 to 0 of Euro-Atlantic blocking, shown for (left) the MERRA2 reanalysis and (right) the CTL run.

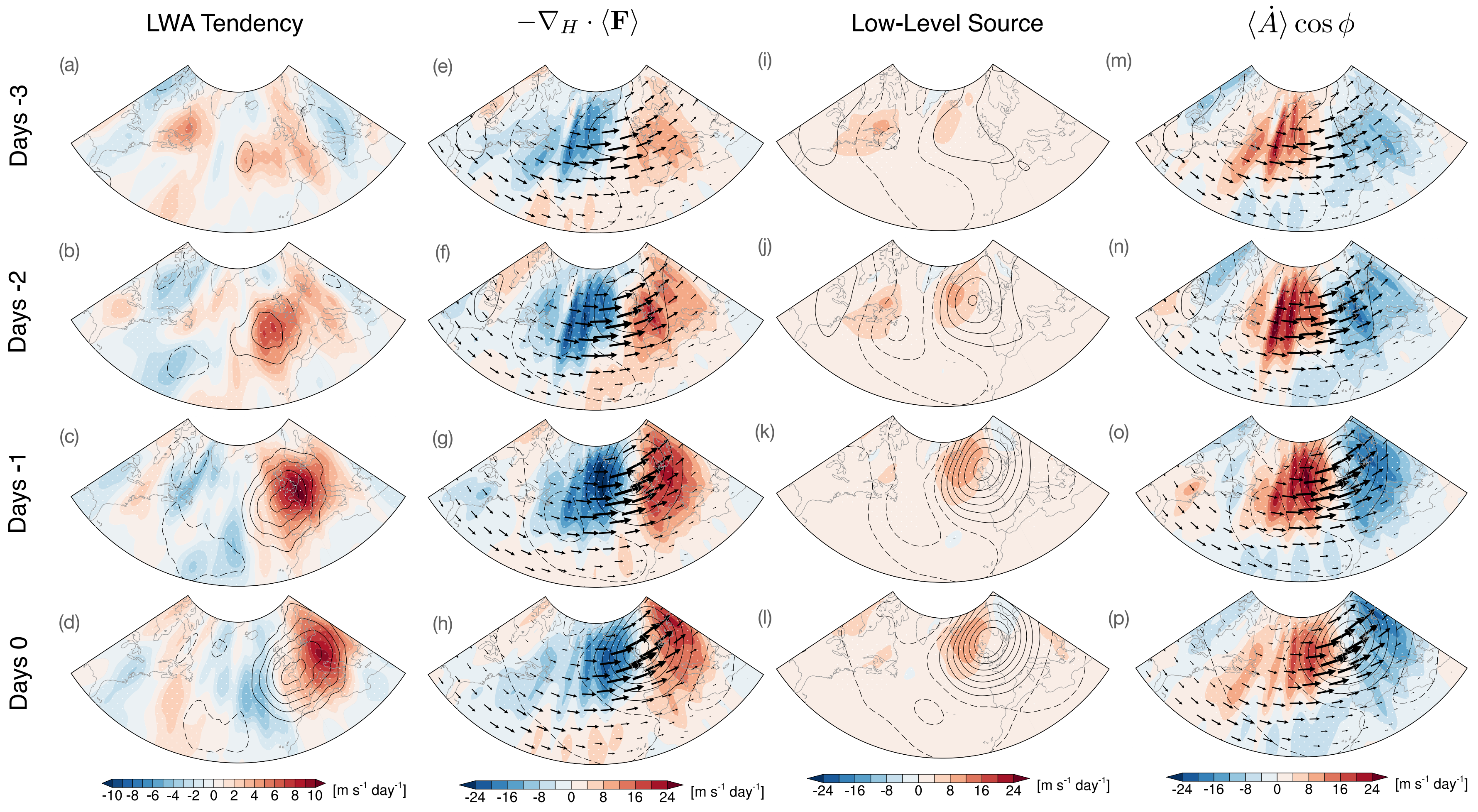

Fig S2 Development of Euro-Atlantic blocking in the reanalysis based on column LWA budget. (a-d) Composites of column LWA budget terms (shading, unit: m s^{-1} day^{-1}) from the onset to the mature stage of the block (days -3 to day 0) from MERRA2 reanalysis. Each column from left to right corresponds to each of LWA tendency terms: (a-d) net tendency (shading); (e-h) vertically averaged horizontal wave activity flux (arrows; F_λ, F_ϕ) and its convergence (shading; $-\nabla_H \cdot \langle \mathbf{F} \rangle$); (i-l) meridional eddy heat flux at the base of the atmosphere (low-level source); and (m-p) sources and sinks of wave activity ($\langle \dot{A} \rangle \cos\phi$ or residual). The contour lines in panels (a-d) denote LWA anomalies (units: m s^{-1}), while the contour lines in panels (e-p) represent Geopotential height anomalies (units: m, interval: 50 m). The stippling indicates the regions where the anomaly is statistically significant at the 95% level based on a bootstrap test.

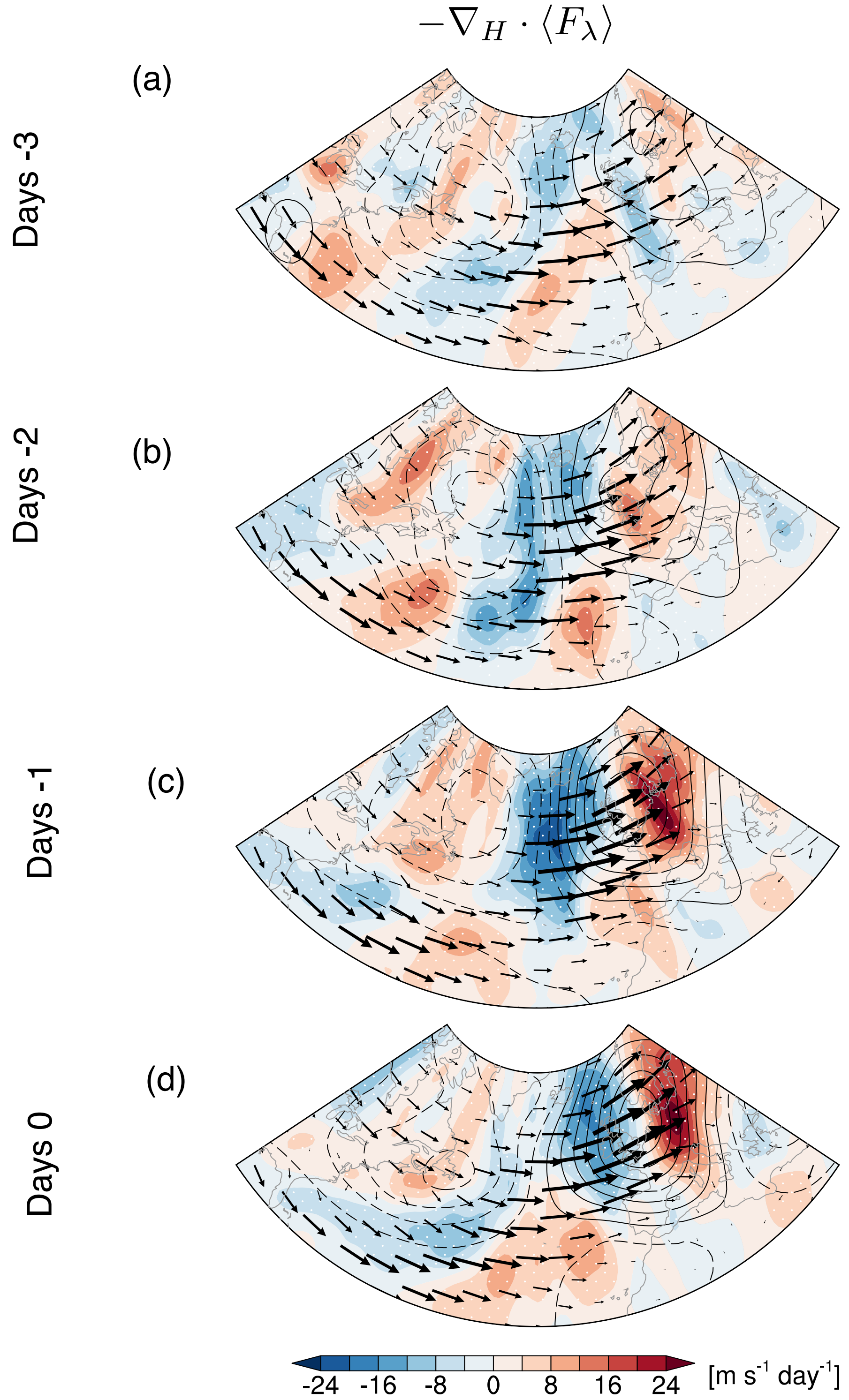

Fig S3 Development of Euro-Atlantic blocking in the CTL based on column LWA budget. (a-d) Composites of column zonal LWA convergence by the nonlinear Stokes term (shading, unit: m s^{-1} day^{-1}) from the onset to the mature stage of the block (days -3 to day 0) from CTL. The contour lines in panels (a-d) represent Geopotential height anomalies (units: m, interval: 50 m). The stippling indicates the regions where the anomaly is statistically significant at the 95% level based on a bootstrap test.

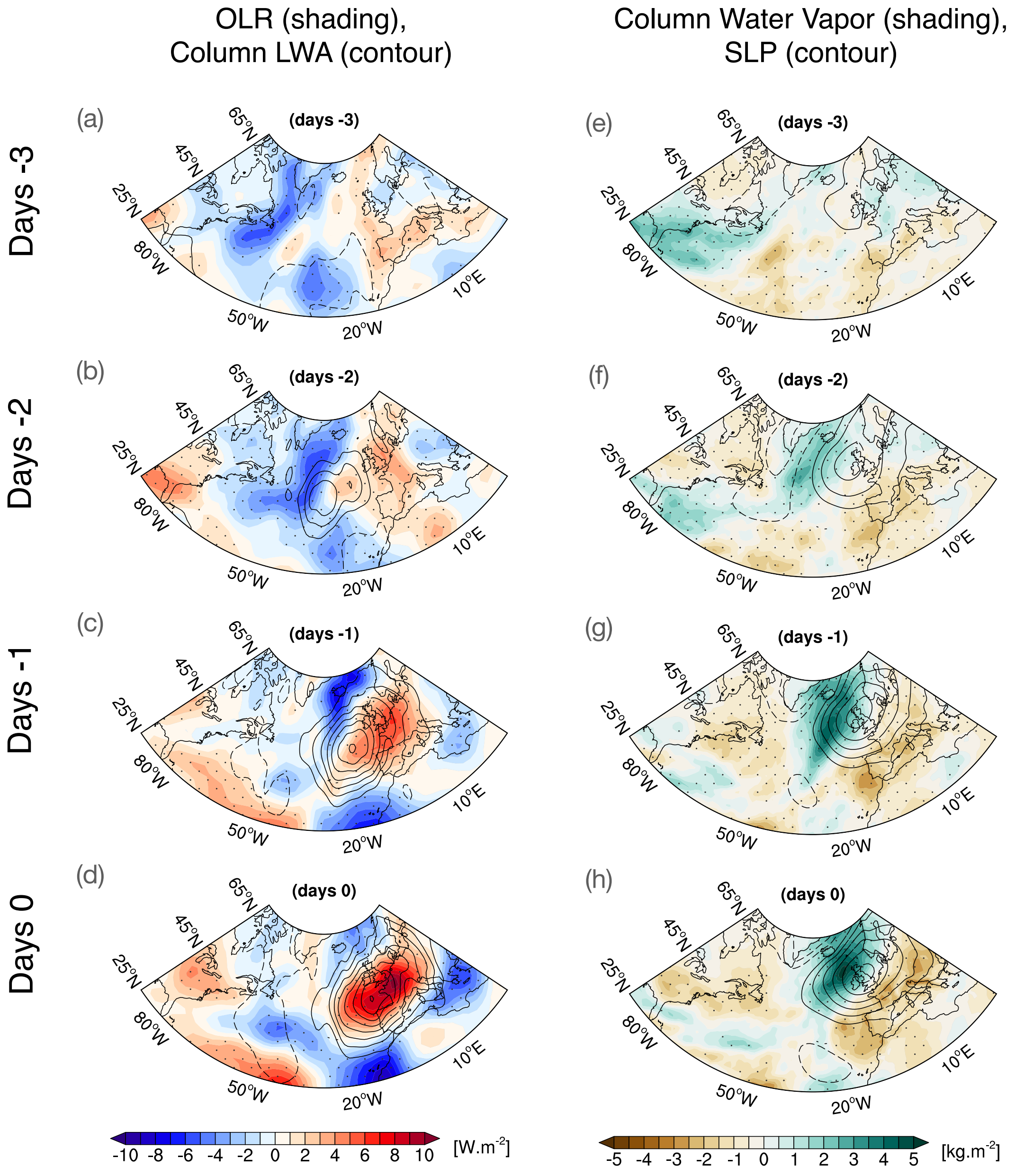

Fig S4 Composite maps of outgoing longwave radiation (OLR) and column water vapor anomalies during the lifecycle of Euro-Atlantic blocking in the reanalysis. (a-d) Composites of OLR (shading, unit: W m^{-2}) from NOAA and column LWA (contour, unit: m s^{-1}) anomalies from the onset to the mature stage of the block (days -3 to day 0) from MERRA2 reanalysis. (e-h) As in (a-d) but for column water vapour (shading, unit: kg m^{-2}) and mean sea-level pressure (contour, unit: hPa, interval: 2 hPa) anomalies. The stippling indicates the regions where the anomaly is statistically significant at the 95% level based on a bootstrap test.

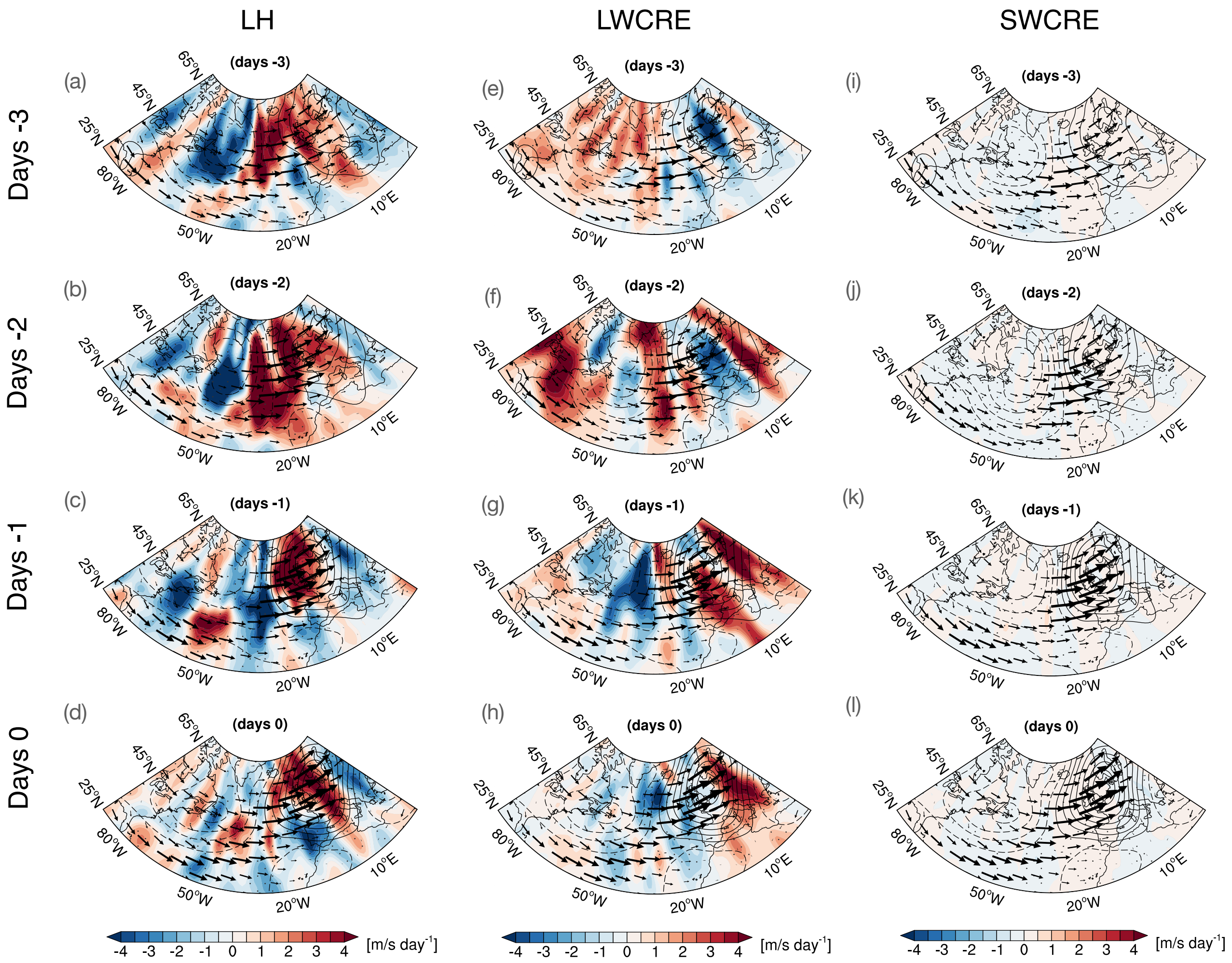

Fig S5 Diabatic source/sink of wave activity during the onset of Euro-Atlantic blocking in CTL. Composite diabatic source/sink of wave activity anomaly (shading, unit: m $s^{-1}day^{-1}$) at 500 hPa in the CTL from the onset to the mature stage of the block (days -3 to day 0). Vectors indicate horizontal wave activity flux (F_λ, F_ϕ). The diabatic source/sink is explicitly calculated using diabatic heating from latent heating (LH), long-wave CRE (LWCRE), and shortwave CRE (SWCRE). The long-wave and short-wave radiation heating during clear-sky (QRLc and QRSc, respectively) are not shown because of the relatively small contribution. The stippling indicates the regions where the anomaly is statistically significant at the 95% level based on a bootstrap test.

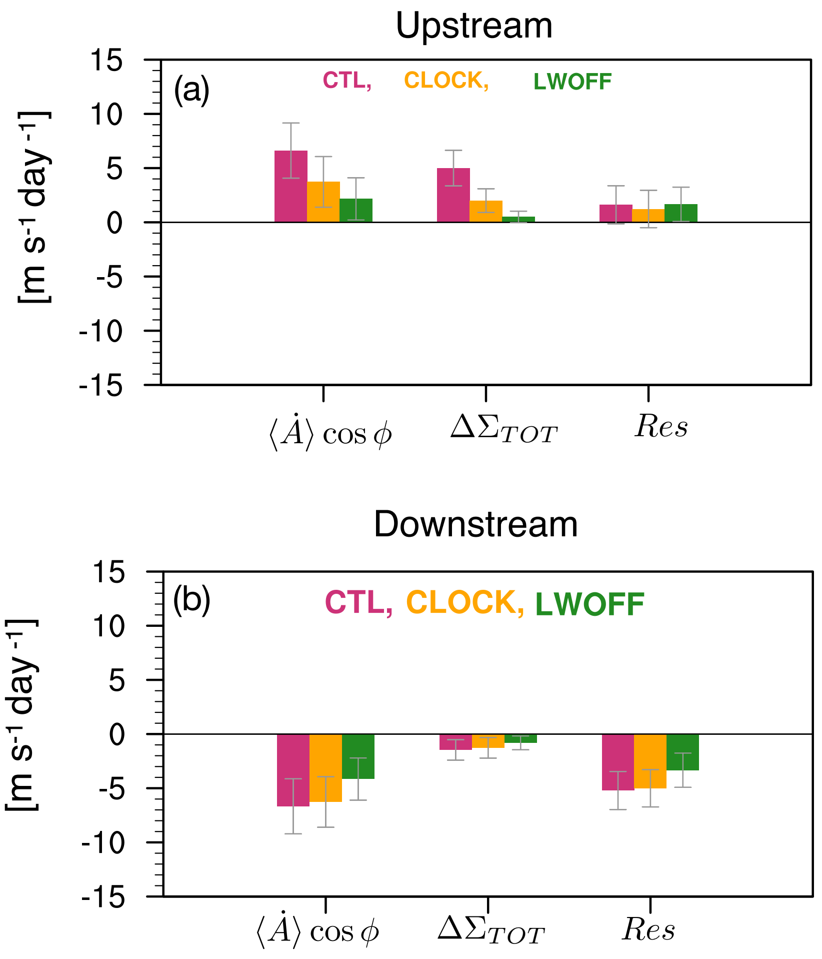

Fig S6 Contribution of non-advective sources and sinks of wave activity during the onset of Euro-Atlantic blocking in E3SM simulations. Non-advective sources and sinks of wave activity ($\langle \dot{A} \rangle \cos\phi$) are decomposed into contributions from total diabatic heating ($\Delta\Sigma_{TOT}$) and residuals (dissipation, surface damping, and error analysis) in units of m s^{-1} day^{-1}, averaged over the upstream region from the onset to the mature stage of the block.

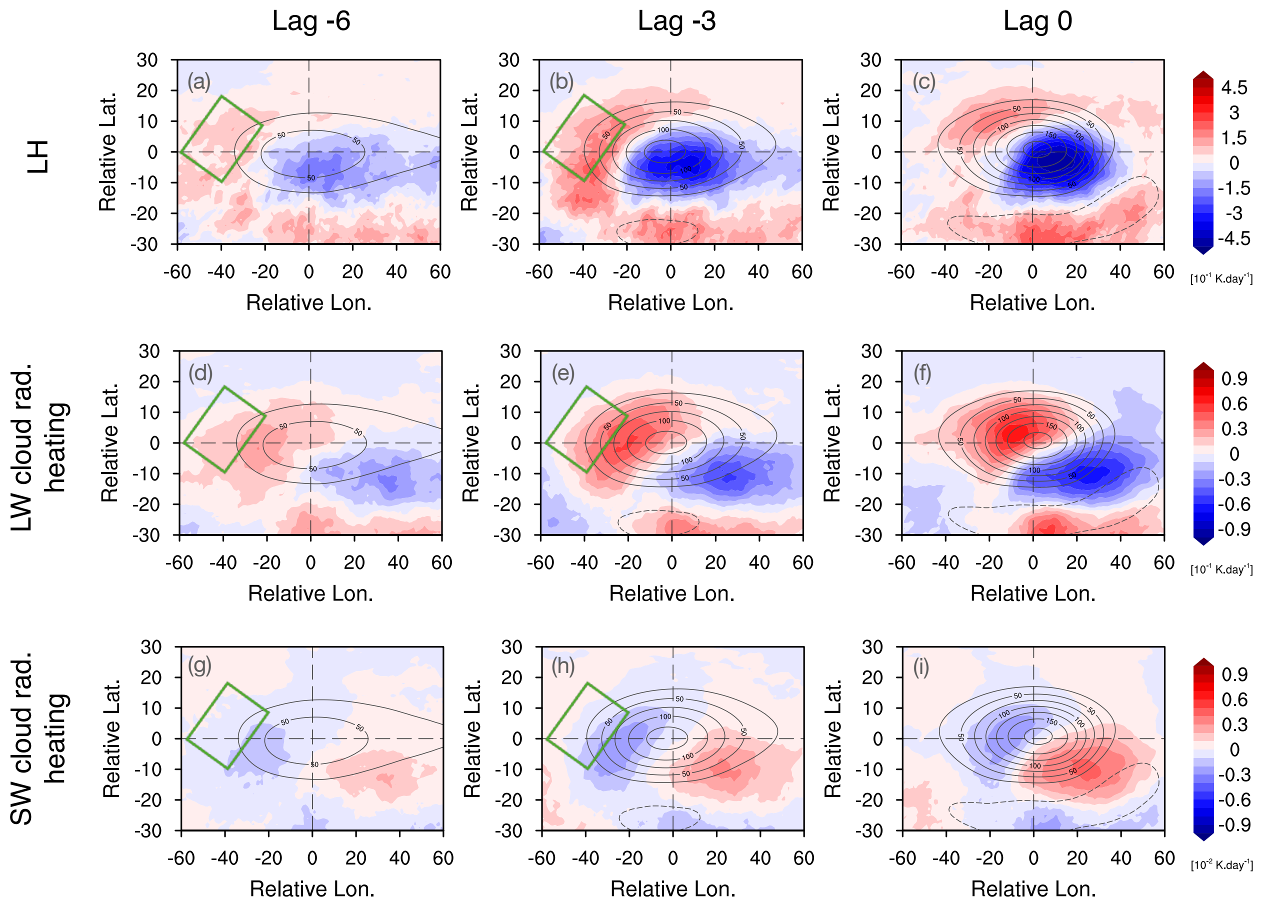

Fig S7 Blocking-relative composites of diabatic heating anomalies from the onset to the mature stage of the block from MERRA2. (a-c) Composite life-cycle of column-averaged pressure-weighted latent heating (LH) anomalies from days -6, -3 and 0. (d-f) As in (a-c) but from LW cloud radiative heating. (g-i) As in (a-c) but from SW cloud radiative heating (shading, unit: x10^{-1} K s^{-1} day^{-1}). Note that the colorbars for LW cloud radiative heating (LWCRE) and SW cloud radiative heating (SWCRE) are scaled down by a factor of five to improve clarity. The contour lines in panels (a-d) represent Geopotential height anomalies (units: m, interval: 25 m). The green square marks the approximate upstream region (west-northwest) of the block, where LH is enhanced.

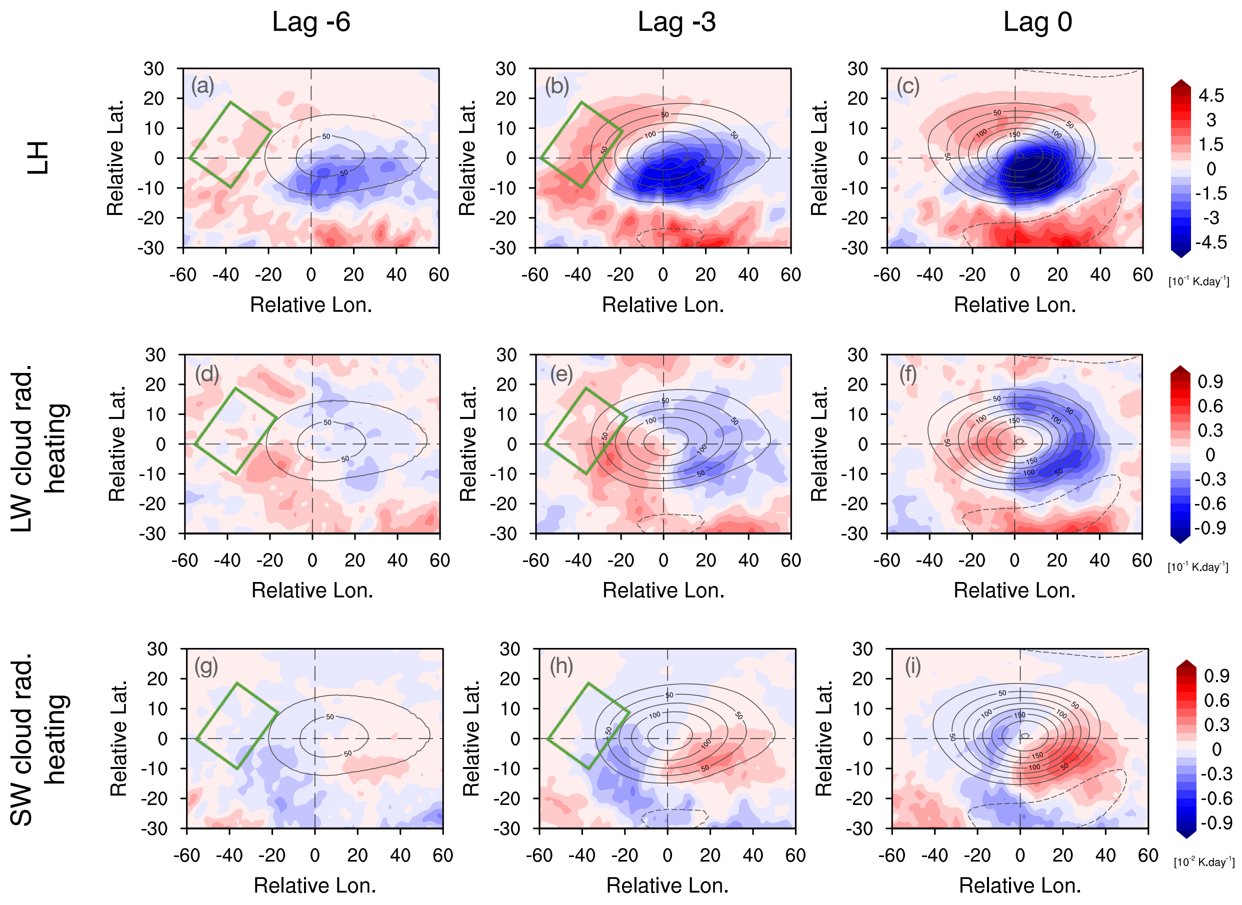

Fig S8 Blocking-relative composites of diabatic heating anomalies from the onset to the mature stage of the block from CTL. (a-c) Composite life-cycle of column-averaged pressure-weighted latent heating (LH) anomalies from days -6, -3 and 0. (d-f) As in (a-c) but from LW cloud radiative heating. (g-i) As in (a-c) but from SW cloud radiative heating (shading, unit: x10^{-1} K s^{-1} day^{-1}). Note that the colorbars for LWCRE and SWCRE are scaled down by a factor of five to improve clarity. The contour lines in panels (a-d) represent Geopotential height anomalies (units: m, interval: 25 m). The green square marks the approximate upstream region (west-northwest) of the block, where LH is enhanced.

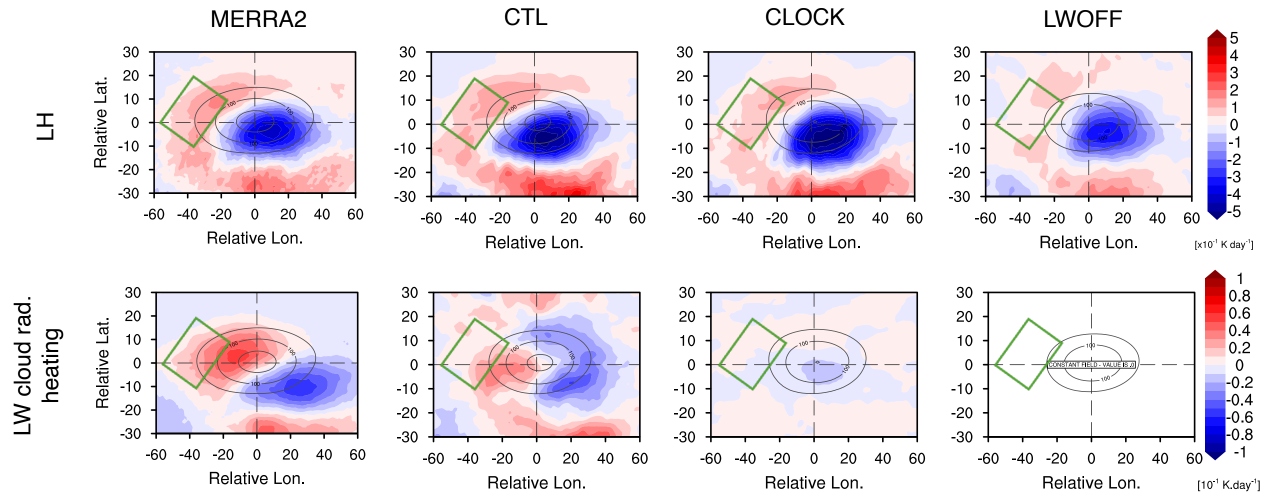

Fig S9 A comparison of blocking-relative composites of diabatic heating in MERRA and model experiments, averaged over the onset period. (a-d) Composites of column-averaged pressure-weighted LH anomalies (shading, unit: x10^{-1} K s^{-1} day^{-1}) averaged from days -3 to -1 for (a) MERRA2, (b) CTL, (c) CLOCK, and (d) LWOFF. (e-h) As in (a-d) but for LW cloud radiative heating anomalies (shading, unit: x10^{-1} K s^{-1} day^{-1}). Note that the colorbar for LW cloud radiative heating is scaled down by a factor of five to improve clarity. The contour lines in panels (a-d) represent Geopotential height anomalies (units: m, interval: 50 m).

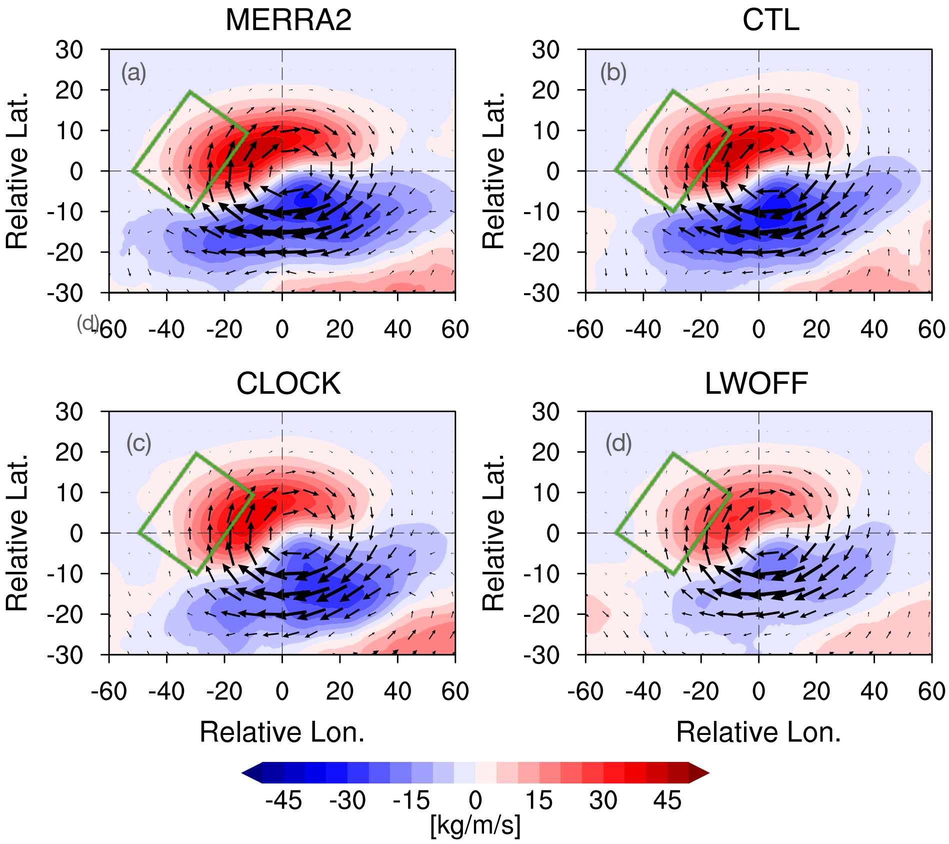

Fig S10 A comparison of blocking-relative composites of IVT in MERRA and model experiments, averaged over the onset period. (a-d) Composites of vertically integrated moisture transport (IVT) anomalies (shading, unit: kg m^{-1} s^{-1}) averaged from days -3 and -1 for (a) MERRA2, (b) CTL, (c) CLOCK, and (d) LWOFF. Vectors represent column water vapor flux.

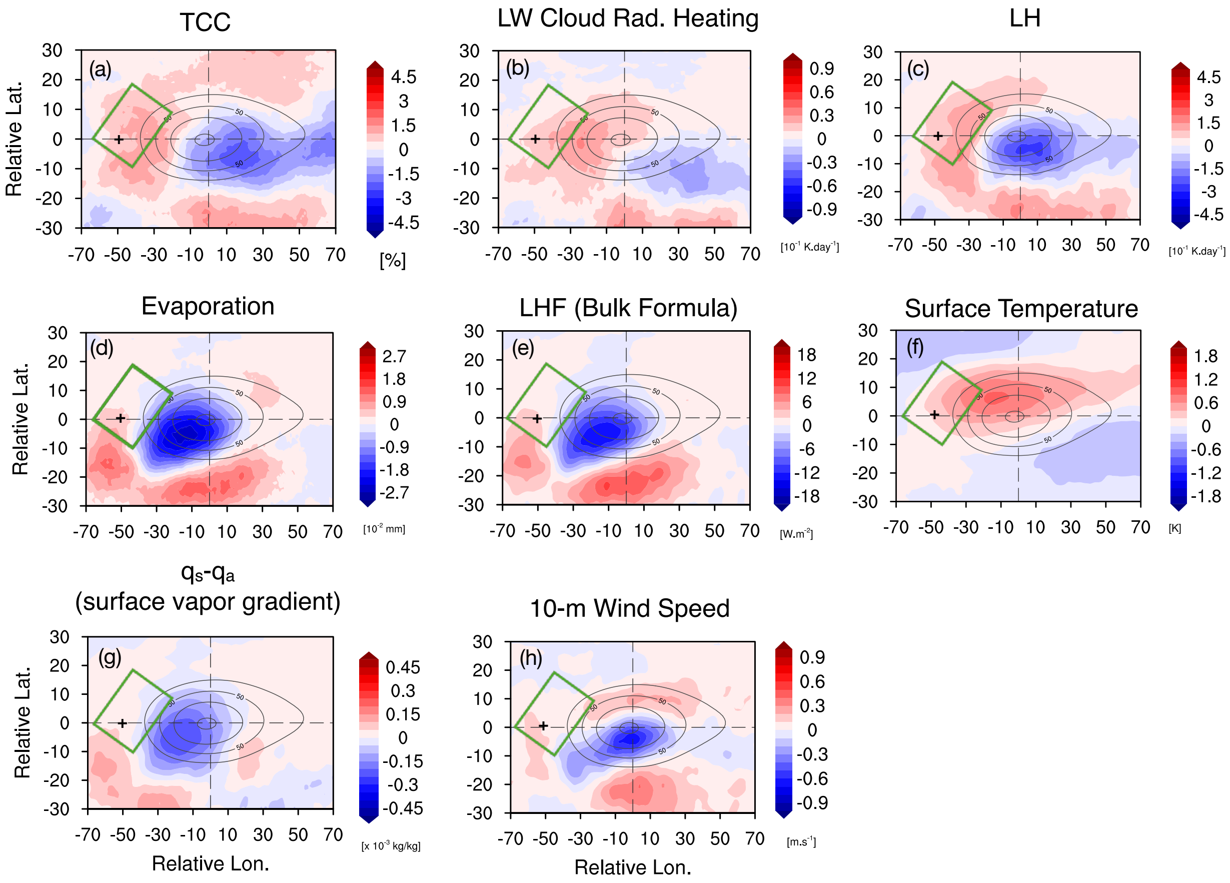

Fig S11 Blocking-relative composites of cloud, radiation, and surface thermodynamic variables averaged from days -6 to -1 before the mature stage of the block from MERRA2. (a) Total cloud cover (TCC; shading, unit: %). (b) Longwave cloud radiative heating (shading, unit: $\times 10^{-1}$ K day^{-1}). (c) Latent heating (shading, unit: $\times 10^{-1}$ K day^{-1}). (d) Evaporation (shading, unit: $\times 10^{-2}$ mm). (e) Latent heat flux from the bulk formula (shading, unit: W m^{-2}). (f) Surface temperature (shading, unit: K). (g) Surface vapor gradient $q_s - q_a$ (shading, unit: $\times 10^{-3}$ kg kg^{-1}). (h) 10-m wind speed (shading, unit: m s^{-1}). Contour lines in all panels represent geopotential height anomalies (units: m; contour interval: 25 m). The green square marks the approximate upstream region (west-northwest of the block center), where latent heating is enhanced. Note that In MERRA2, downward fluxes are positive; evaporation is multiplied by -1 so positive values indicate evaporation.

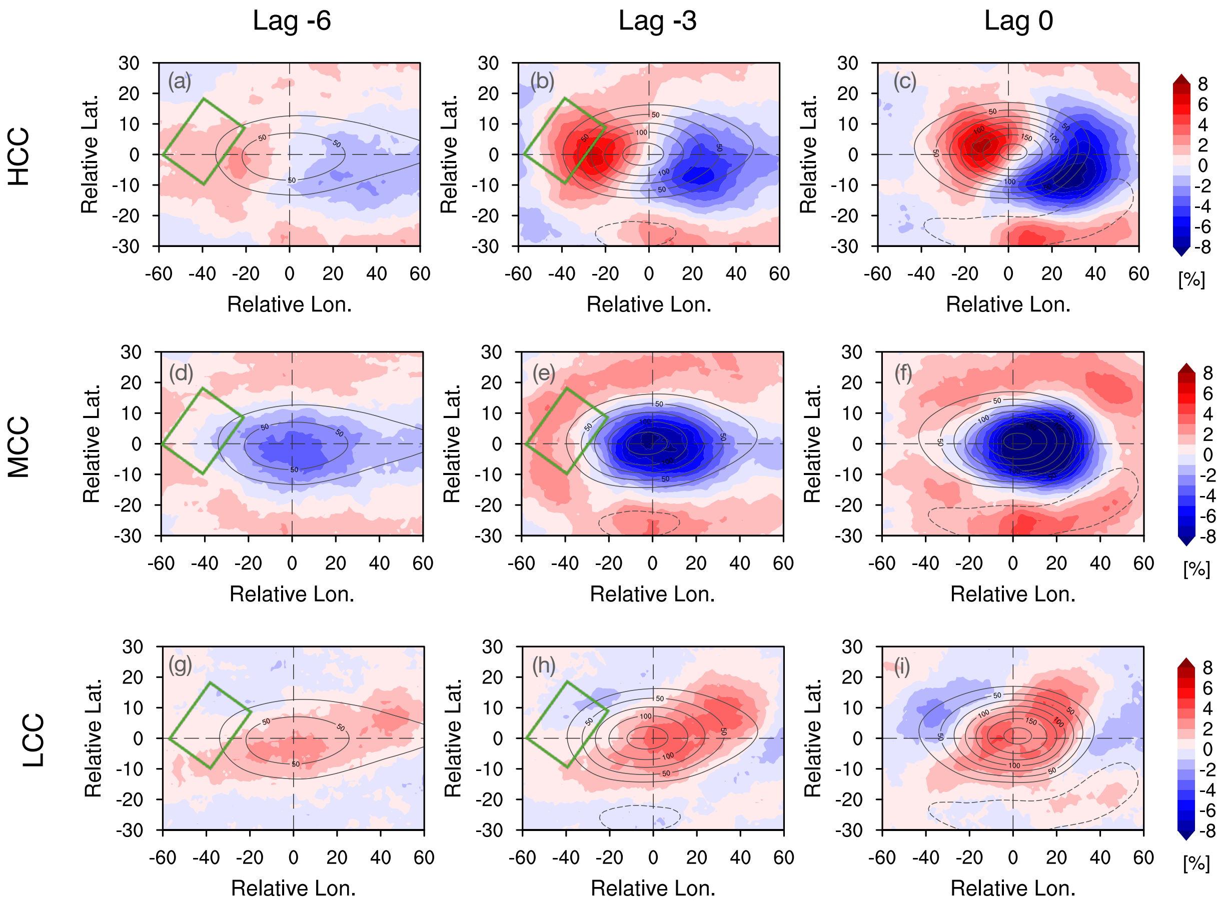

Fig S12 Blocking-relative composites of cloud cover fraction at different altitudes from the onset to the mature stage of the block in MERRA-2. (a-c) Composite life-cycle of high cloud cover (HCC, altitude: >6 km) anomalies from days -6, -3 and 0. (d-f) As in (a-c) but for medium cloud cover (MCC, altitude: 3-6 km). (g-i) As in (a-c) but for low cloud cover (LCC, altitude: 0-3 km) (shading, unit: %). The contour lines in panels (a-d) represent Geopotential height anomalies (units: m, interval: 25 m). The green square marks the approximate upstream region (west-northwest) of the block, where LH is enhanced.

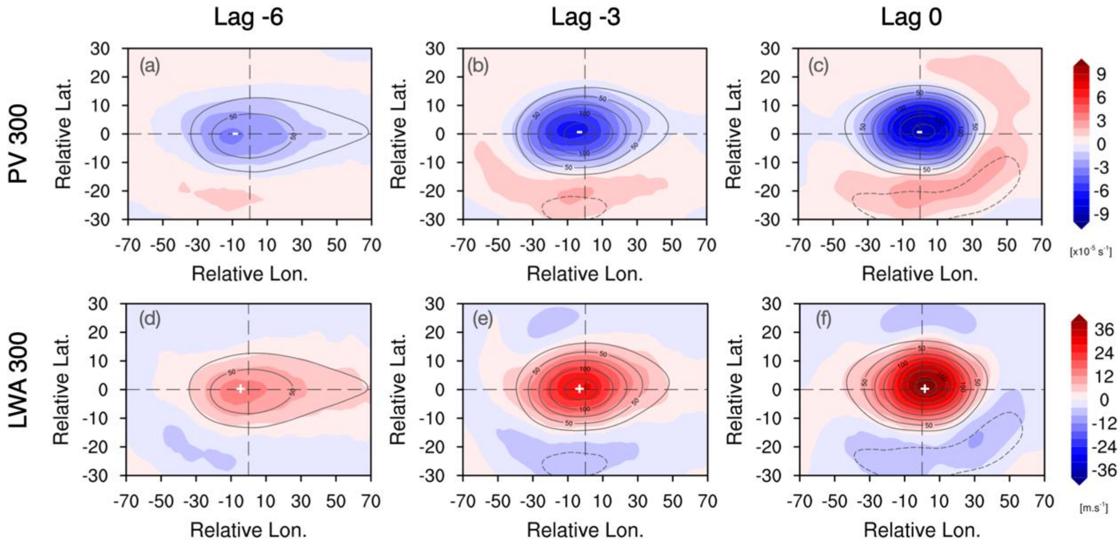

Fig S13 Blocking-relative composite lifecycle of (a-c) 300-hPa PV anomalies (shading) and (d-f) 300-hPa LWA anomalies (shading) from MERRA. Contours show Z500 anomalies. The evolution of upper-level LWA anomalies closely follows the development of the blocking pattern and the associated upper-level negative PV anomalies, illustrating how the finite-amplitude LWA framework provides a complementary perspective on blocking onset. It is noteworthy that both the column-integrated LWA and its budget are dominated by upper-level features, as wave amplitude is largest in the upper troposphere.
